# Supplementary material for: Novel Driver Strength Index highlights important cancer genes in TCGA PanCanAtlas patients
Source: PeerJ. 2022 Aug 11;10:e13860. doi: 10.7717/peerj.13860 (PMC9375969; doi:10.7717/peerj.13860)
Supplement: Supplemental Information 2 [file peerj-10-13860-s002.zip › COHORTS/genes plots/detailed/2021_8_16_14_9_distribution_events_detailed_22.pdf]

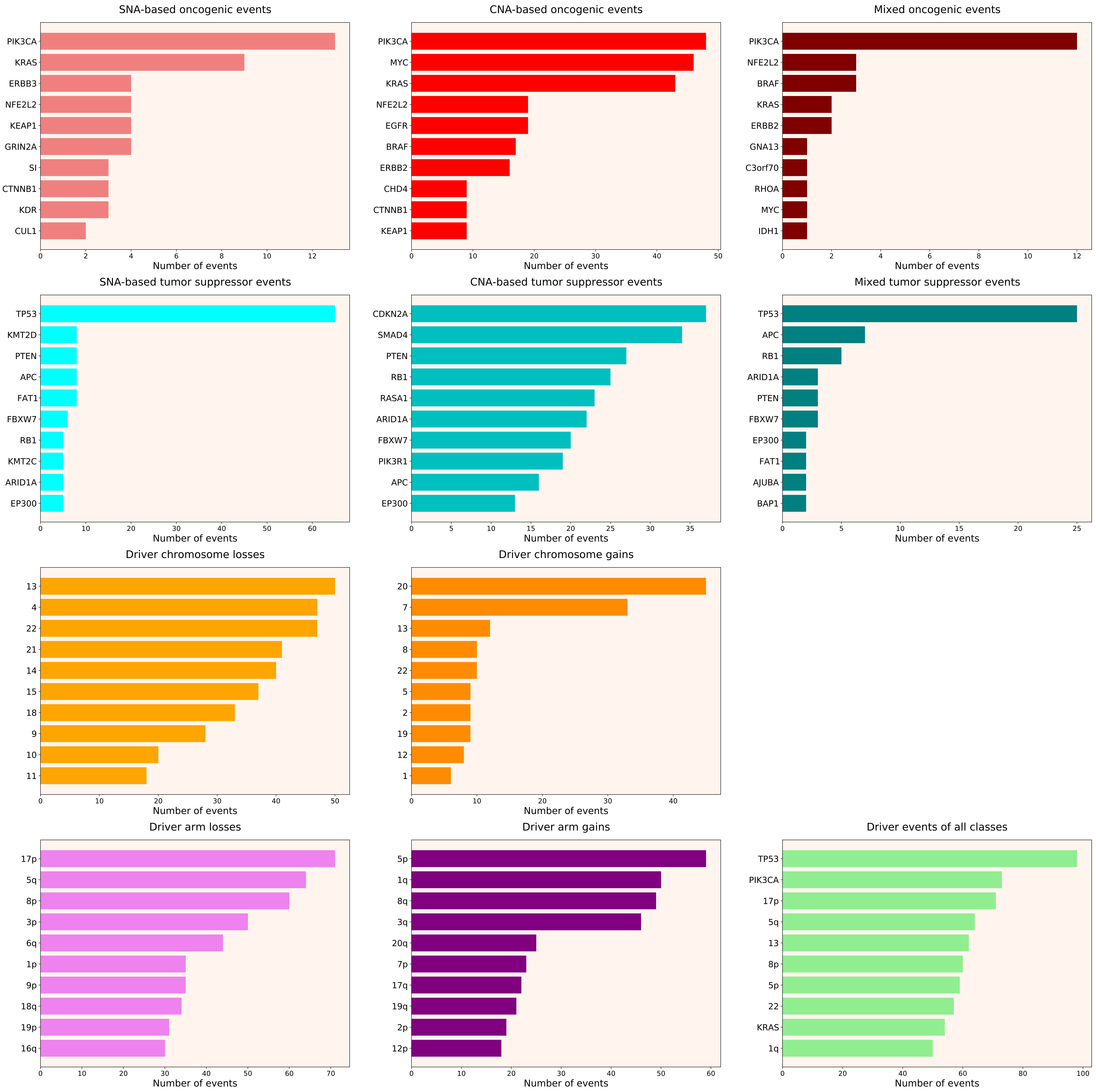
SNA-based oncogenic events
PIK3CA
KRAS
ERBB3
NFE2L2
KEAP1
GRIN2A
SI
CTNNB1
KDR
CUL1
0 2 4 6 8 10 12
Number of events
CNA-based oncogenic events
PIK3CA
MYC
KRAS
NFE2L2
EGFR
BRAF
ERBB2
CHD4
CTNNB1
KEAP1
0 10 20 30 40 50
Number of events
Mixed oncogenic events
PIK3CA
NFE2L2
BRAF
KRAS
ERBB2
GNA13
C3orf70
RHOA
MYC
IDH1
0 2 4 6 8 10 12
Number of events
SNA-based tumor suppressor events
TP53
KMT2D
PTEN
APC
FAT1
FBXW7
RB1
KMT2C
ARID1A
EP300
0 10 20 30 40 50 60
Number of events
CNA-based tumor suppressor events
CDKN2A
SMAD4
PTEN
RB1
RASA1
ARID1A
FBXW7
PIK3R1
APC
EP300
0 5 10 15 20 25 30 35
Number of events
Mixed tumor suppressor events
TP53
APC
RB1
ARID1A
PTEN
FBXW7
EP300
FAT1
AJUBA
BAP1
0 5 10 15 20 25
Number of events
Driver chromosome losses
13
4
22
21
14
15
18
9
10
11
0 10 20 30 40 50
Number of events
Driver chromosome gains
20
7
13
8
22
5
2
19
12
1
0 10 20 30 40
Number of events
Driver arm losses
17p
5q
8p
3p
6q
1p
9p
18q
19p
16q
0 10 20 30 40 50 60 70
Number of events
Driver arm gains
5p
1q
8q
3q
20q
7p
17q
19q
2p
12p
0 10 20 30 40 50 60
Number of events
Driver events of all classes
TP53
PIK3CA
17p
5q
13
8p
5p
22
KRAS
1q
0 20 40 60 80 100
Number of events
